# Monoclinic polymorph of 3,7-dimethyl-1-(5-oxohex­yl)-3,7-dihydro-1*H*-purine-2,6-dione

**DOI:** 10.1107/S1600536811040232

**Published:** 2011-10-05

**Authors:** Dmitrijs Stepanovs, Anatoly Mishnev

**Affiliations:** a Latvian Institute of Organic Synthesis, 21 Aizkraukles Street, Riga, LV-1006, Latvia

## Abstract

The structure of the title compound, pentoxifylline, C_13_H_18_N_4_O_3_, has been previously characterized as a triclinic polymorph [Pavelčík *et al.* (1989 ▶). *Acta Cryst*. C**45**, 836–837]. We have discovered the monoclinic form. There are no strong hydrogen bonds in the crystal structure, rather, moderate C—H⋯O hydrogen bonds are present, which serve to stabilize the three-dimensional architecture.

## Related literature

For general background to pentoxifylline, see Dettelbach & Aviado (1985 ▶). For the structure and the nature of the hydrogen bonding in the triclinic polymorph, see: Pavelčík *et al.* (1989 ▶); Gilli (2002 ▶).
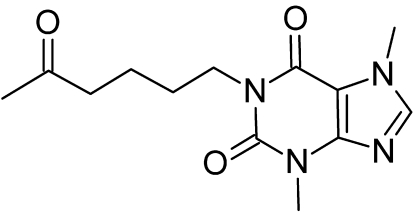


## Experimental

### 

#### Crystal data

C_13_H_18_N_4_O_3_
*M* *_r_* = 278.31
Monoclinic, 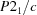

*a* = 9.743 (6) Å
*b* = 17.410 (8) Å
*c* = 7.956 (3) Å
β = 90.89 (2)°
*V* = 1349.4 (12) Å^3^
*Z* = 4
Mo *K*α radiation
μ = 0.10 mm^−1^
*T* = 190 K
0.40 × 0.30 × 0.05 mm

#### Data collection

Nonius KappaCCD diffractometer
5073 measured reflections
3103 independent reflections
1817 reflections with *I* > 2σ(*I*)
*R*_int_ = 0.053

#### Refinement

*R*[*F* ^2^ > 2σ(*F* ^2^)] = 0.066
*wR*(*F* ^2^) = 0.163
*S* = 1.01
3065 reflections
184 parameters
H-atom parameters constrained
Δρ_max_ = 0.24 e Å^−3^
Δρ_min_ = −0.20 e Å^−3^

### 

Data collection: *KappaCCD Server Software* (Nonius, 1997 ▶); cell refinement: *HKL SCALEPACK* (Otwinovski & Minor, 1997 ▶); data reduction: *HKL DENZO* (Otwinovski & Minor, 1997 ▶) and *SCALEPACK*; program(s) used to solve structure: *SHELXS97* (Sheldrick, 2008 ▶); program(s) used to refine structure: *SHELXL97* (Sheldrick, 2008 ▶); molecular graphics: *ORTEP-3* (Farrugia, 1997 ▶); software used to prepare material for publication: *SHELXL97*.

## Supplementary Material

Crystal structure: contains datablock(s) I, global. DOI: 10.1107/S1600536811040232/tk2793sup1.cif

Structure factors: contains datablock(s) I. DOI: 10.1107/S1600536811040232/tk2793Isup2.hkl

Supplementary material file. DOI: 10.1107/S1600536811040232/tk2793Isup3.cml

Additional supplementary materials:  crystallographic information; 3D view; checkCIF report

## Figures and Tables

**Table 1:** Hydrogen-bond geometry (Å, °)

*D*—H⋯*A*	*D*—H	H⋯*A*	*D*⋯*A*	*D*—H⋯*A*
C2—H2⋯O18^i^	0.93	2.39	3.206 (4)	147
C15—H15*B*⋯O19^ii^	0.96	2.60	3.439 (4)	147
C16—H16*A*⋯O18^i^	0.96	2.55	3.395 (4)	148

Symmetry codes: (i) 
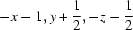
; (ii) 
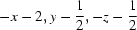
.

**Fig. 1.**
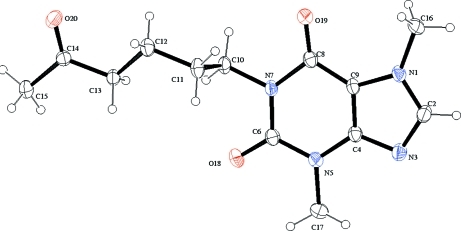
The molecular structure of the title compound, II, showing 50% probability ellipsoids and hydrogen atoms are shown as small spheres of arbitrary radii.

## supplementary crystallographic information

### Comment

The background to pentoxifylline has been summarized (Dettelbach & Aviado, 1985). The polymorph structure II differs from the known form I Pavelčík *et al.*, 1989; Gilli, 2002) in their molecular conformation and the packing of the molecules in the lattice. The bond lengths of forms I and II are close to their standard values, but torsion angle N7—C10—C11—C12 (75.6 (3)° for I and 175.8 (2)° for II) and some bond angles significantly differ: N7—C10—C11 (114.2 (2)° for I and 111.9 (2)° for II), C10—C11—C12 (115.4 (2)° for I and 115.4 (2)° for II), C11—C12—C13 (111.0 (2)° for I and 114.1 (2)° for II). In the crystal structure of II, the molecules are connected by means of C—H···O hydrogen bonds, Table 1.

### Experimental

Title compound was obtained by recrystallization of Trental tablets, produced by Sanofi-Aventis Deutschland GmbH. Crystals were grown by slow evaporation from dichloromethane at temperature range 308–315 K.

### Refinement

All hydrogen atoms were positioned geometrically with C—H distances ranging from 0.93 to 0.97 Å and refined as riding on their parent atoms with *U*_iso_ (H) = 1.5*U*_eq_ (C) for methyl groups and *U*_iso_ (H) = 1.2*U*_eq_ (C) for others.

### Crystal data

**Table:**

C_13_H_18_N_4_O_3_	*D*_x_ = 1.370 Mg m^−^^3^
*M**_r_* = 278.31	Melting point: 365 K
Monoclinic, *P*2_1_/*c*	Mo *K*α radiation, λ = 0.71073 Å
*a* = 9.743 (6) Å	Cell parameters from 6273 reflections
*b* = 17.410 (8) Å	θ = 1.0–27.5°
*c* = 7.956 (3) Å	µ = 0.10 mm^−^^1^
β = 90.89 (2)°	*T* = 190 K
*V* = 1349.4 (12) Å^3^	Plate, colourless
*Z* = 4	0.4 × 0.3 × 0.05 mm
*F*(000) = 592	

### Data collection

**Table:**

Nonius KappaCCD diffractometer	1817 reflections with *I* > 2σ(*I*)
Radiation source: fine-focus sealed tube	*R*_int_ = 0.053
graphite	θ_max_ = 27.5°, θ_min_ = 2.4°
CCD scans	*h* = −12→12
5073 measured reflections	*k* = −22→19
3065 independent reflections	*l* = −10→10

### Refinement

**Table:**

Refinement on *F*^2^	Primary atom site location: structure-invariant direct methods
Least-squares matrix: full	Secondary atom site location: difference Fourier map
*R*[*F*^2^ > 2σ(*F*^2^)] = 0.066	Hydrogen site location: inferred from neighbouring sites
*wR*(*F*^2^) = 0.150	H-atom parameters constrained
*S* = 1.02	*w* = 1/[σ^2^(*F*_o_^2^) + (0.0525*P*)^2^ + 0.5672*P*] where *P* = (*F*_o_^2^ + 2*F*_c_^2^)/3
3065 reflections	(Δ/σ)_max_ = 0.004
184 parameters	Δρ_max_ = 0.24 e Å^−^^3^
0 restraints	Δρ_min_ = −0.20 e Å^−^^3^

### Special details

**Table:**

Geometry. All e.s.d.'s (except the e.s.d. in the dihedral angle between two l.s. planes) are estimated using the full covariance matrix. The cell e.s.d.'s are taken into account individually in the estimation of e.s.d.'s in distances, angles and torsion angles; correlations between e.s.d.'s in cell parameters are only used when they are defined by crystal symmetry. An approximate (isotropic) treatment of cell e.s.d.'s is used for estimating e.s.d.'s involving l.s. planes.
Refinement. Refinement of *F*^2^ against ALL reflections. The weighted *R*-factor *wR* and goodness of fit *S* are based on *F*^2^, conventional *R*-factors *R* are based on *F*, with *F* set to zero for negative *F*^2^. The threshold expression of *F*^2^ > σ(*F*^2^) is used only for calculating *R*-factors(gt) *etc*. and is not relevant to the choice of reflections for refinement. *R*-factors based on *F*^2^ are statistically about twice as large as those based on *F*, and *R*- factors based on ALL data will be even larger.

### Fractional atomic coordinates and isotropic or equivalent isotropic displacement parameters (Å^2^)

**Table:**

	*x*	*y*	*z*	*U*_iso_*/*U*_eq_	
O19	−0.79244 (18)	0.03718 (9)	−0.4841 (2)	0.0407 (5)	
O18	−0.54702 (19)	−0.15692 (9)	−0.2345 (2)	0.0431 (5)	
N7	−0.6688 (2)	−0.05902 (10)	−0.3550 (2)	0.0314 (5)	
O20	−1.1444 (2)	−0.31616 (11)	−0.4130 (3)	0.0550 (6)	
N1	−0.5641 (2)	0.14522 (11)	−0.3470 (2)	0.0337 (5)	
N5	−0.4583 (2)	−0.03670 (11)	−0.2089 (2)	0.0350 (5)	
C9	−0.5831 (2)	0.06649 (13)	−0.3380 (3)	0.0304 (5)	
N3	−0.3878 (2)	0.09676 (12)	−0.1963 (3)	0.0380 (5)	
C4	−0.4742 (2)	0.04006 (13)	−0.2463 (3)	0.0319 (6)	
C10	−0.7740 (3)	−0.11505 (13)	−0.4085 (3)	0.0351 (6)	
H10A	−0.7303	−0.1639	−0.4317	0.042*	
H10B	−0.8180	−0.0973	−0.5116	0.042*	
C6	−0.5568 (3)	−0.08839 (14)	−0.2631 (3)	0.0331 (6)	
C13	−0.9288 (3)	−0.26965 (13)	−0.3170 (3)	0.0323 (6)	
H13A	−0.8532	−0.2731	−0.3946	0.039*	
H13B	−0.8920	−0.2790	−0.2049	0.039*	
C14	−1.0310 (3)	−0.33177 (14)	−0.3593 (3)	0.0326 (6)	
C12	−0.9861 (2)	−0.18878 (13)	−0.3236 (3)	0.0361 (6)	
H12A	−1.0633	−0.1856	−0.2484	0.043*	
H12B	−1.0203	−0.1787	−0.4366	0.043*	
C15	−0.9876 (3)	−0.41325 (14)	−0.3319 (3)	0.0412 (6)	
H15A	−1.0331	−0.4457	−0.4128	0.062*	
H15B	−1.0117	−0.4290	−0.2206	0.062*	
H15C	−0.8900	−0.4173	−0.3446	0.062*	
C17	−0.3361 (3)	−0.06489 (15)	−0.1159 (3)	0.0427 (6)	
H17A	−0.2675	−0.0805	−0.1942	0.064*	
H17B	−0.3612	−0.1079	−0.0474	0.064*	
H17C	−0.3002	−0.0246	−0.0456	0.064*	
C8	−0.6905 (3)	0.01842 (13)	−0.4012 (3)	0.0324 (6)	
C11	−0.8821 (3)	−0.12644 (14)	−0.2754 (3)	0.0388 (6)	
H11A	−0.9303	−0.0784	−0.2583	0.047*	
H11B	−0.8373	−0.1403	−0.1700	0.047*	
C16	−0.6522 (3)	0.20142 (14)	−0.4321 (3)	0.0420 (6)	
H16A	−0.6097	0.2511	−0.4268	0.063*	
H16B	−0.7394	0.2034	−0.3778	0.063*	
H16C	−0.6653	0.1867	−0.5475	0.063*	
C2	−0.4478 (3)	0.15899 (15)	−0.2608 (3)	0.0386 (6)	
H2	−0.4118	0.2081	−0.2469	0.046*	

### Atomic displacement parameters (Å^2^)

**Table:**

	*U*^11^	*U*^22^	*U*^33^	*U*^12^	*U*^13^	*U*^23^
O19	0.0381 (11)	0.0348 (10)	0.0490 (10)	−0.0019 (8)	−0.0061 (8)	0.0061 (8)
O18	0.0530 (12)	0.0232 (9)	0.0530 (11)	0.0008 (8)	−0.0011 (8)	0.0021 (8)
N7	0.0359 (12)	0.0224 (10)	0.0358 (11)	−0.0046 (9)	0.0003 (8)	−0.0003 (8)
O20	0.0438 (13)	0.0385 (11)	0.0818 (14)	−0.0051 (9)	−0.0226 (10)	−0.0014 (10)
N1	0.0327 (12)	0.0231 (10)	0.0452 (12)	0.0017 (9)	0.0017 (9)	0.0010 (8)
N5	0.0334 (12)	0.0288 (11)	0.0428 (12)	0.0007 (9)	−0.0018 (9)	0.0033 (9)
C9	0.0322 (13)	0.0217 (11)	0.0374 (13)	−0.0010 (10)	0.0039 (10)	0.0004 (10)
N3	0.0362 (12)	0.0304 (11)	0.0473 (12)	−0.0030 (10)	−0.0003 (9)	−0.0037 (9)
C4	0.0313 (14)	0.0261 (12)	0.0384 (13)	0.0001 (11)	0.0032 (10)	−0.0009 (10)
C10	0.0425 (15)	0.0245 (12)	0.0383 (13)	−0.0080 (11)	0.0011 (11)	−0.0020 (10)
C6	0.0370 (14)	0.0279 (13)	0.0346 (13)	0.0003 (11)	0.0044 (10)	−0.0021 (10)
C13	0.0356 (14)	0.0267 (12)	0.0345 (12)	−0.0052 (10)	−0.0001 (10)	0.0001 (10)
C14	0.0366 (15)	0.0322 (13)	0.0290 (12)	−0.0029 (11)	−0.0008 (10)	−0.0002 (10)
C12	0.0335 (14)	0.0284 (13)	0.0465 (14)	−0.0009 (11)	0.0060 (11)	−0.0015 (11)
C15	0.0443 (16)	0.0282 (13)	0.0510 (15)	−0.0050 (12)	−0.0026 (12)	0.0002 (11)
C17	0.0404 (16)	0.0372 (14)	0.0503 (16)	0.0079 (12)	−0.0051 (12)	0.0051 (12)
C8	0.0332 (14)	0.0294 (13)	0.0347 (13)	0.0002 (11)	0.0061 (11)	0.0011 (10)
C11	0.0453 (16)	0.0253 (12)	0.0459 (15)	−0.0048 (11)	0.0095 (11)	−0.0065 (11)
C16	0.0392 (15)	0.0289 (14)	0.0578 (16)	0.0077 (11)	−0.0040 (12)	0.0066 (11)
C2	0.0322 (15)	0.0328 (14)	0.0509 (15)	−0.0039 (11)	−0.0013 (11)	−0.0039 (12)

### Geometric parameters (Å, °)

**Table:**

O19—C8	1.228 (3)	C13—C12	1.515 (3)
O18—C6	1.218 (3)	C13—H13A	0.9700
N7—C6	1.401 (3)	C13—H13B	0.9700
N7—C8	1.413 (3)	C14—C15	1.495 (3)
N7—C10	1.473 (3)	C12—C11	1.530 (3)
O20—C14	1.209 (3)	C12—H12A	0.9700
N1—C2	1.337 (3)	C12—H12B	0.9700
N1—C9	1.385 (3)	C15—H15A	0.9600
N1—C16	1.461 (3)	C15—H15B	0.9600
N5—C4	1.377 (3)	C15—H15C	0.9600
N5—C6	1.380 (3)	C17—H17A	0.9600
N5—C17	1.476 (3)	C17—H17B	0.9600
C9—C4	1.359 (3)	C17—H17C	0.9600
C9—C8	1.426 (3)	C11—H11A	0.9700
N3—C2	1.330 (3)	C11—H11B	0.9700
N3—C4	1.353 (3)	C16—H16A	0.9600
C10—C11	1.518 (3)	C16—H16B	0.9600
C10—H10A	0.9700	C16—H16C	0.9600
C10—H10B	0.9700	C2—H2	0.9300
C13—C14	1.505 (3)		
			
C6—N7—C8	126.6 (2)	C13—C12—H12A	108.7
C6—N7—C10	116.24 (19)	C11—C12—H12A	108.7
C8—N7—C10	117.1 (2)	C13—C12—H12B	108.7
C2—N1—C9	105.3 (2)	C11—C12—H12B	108.7
C2—N1—C16	127.2 (2)	H12A—C12—H12B	107.6
C9—N1—C16	127.5 (2)	C14—C15—H15A	109.5
C4—N5—C6	119.3 (2)	C14—C15—H15B	109.5
C4—N5—C17	121.2 (2)	H15A—C15—H15B	109.5
C6—N5—C17	119.4 (2)	C14—C15—H15C	109.5
C4—C9—N1	105.0 (2)	H15A—C15—H15C	109.5
C4—C9—C8	123.6 (2)	H15B—C15—H15C	109.5
N1—C9—C8	131.4 (2)	N5—C17—H17A	109.5
C2—N3—C4	102.3 (2)	N5—C17—H17B	109.5
N3—C4—C9	112.8 (2)	H17A—C17—H17B	109.5
N3—C4—N5	125.2 (2)	N5—C17—H17C	109.5
C9—C4—N5	121.9 (2)	H17A—C17—H17C	109.5
N7—C10—C11	111.85 (19)	H17B—C17—H17C	109.5
N7—C10—H10A	109.2	O19—C8—N7	120.7 (2)
C11—C10—H10A	109.2	O19—C8—C9	128.1 (2)
N7—C10—H10B	109.2	N7—C8—C9	111.2 (2)
C11—C10—H10B	109.2	C10—C11—C12	112.4 (2)
H10A—C10—H10B	107.9	C10—C11—H11A	109.1
O18—C6—N5	121.9 (2)	C12—C11—H11A	109.1
O18—C6—N7	120.9 (2)	C10—C11—H11B	109.1
N5—C6—N7	117.2 (2)	C12—C11—H11B	109.1
C14—C13—C12	114.7 (2)	H11A—C11—H11B	107.8
C14—C13—H13A	108.6	N1—C16—H16A	109.5
C12—C13—H13A	108.6	N1—C16—H16B	109.5
C14—C13—H13B	108.6	H16A—C16—H16B	109.5
C12—C13—H13B	108.6	N1—C16—H16C	109.5
H13A—C13—H13B	107.6	H16A—C16—H16C	109.5
O20—C14—C15	121.3 (2)	H16B—C16—H16C	109.5
O20—C14—C13	121.0 (2)	N3—C2—N1	114.6 (2)
C15—C14—C13	117.6 (2)	N3—C2—H2	122.7
C13—C12—C11	114.1 (2)	N1—C2—H2	122.7
			
C2—N1—C9—C4	−0.3 (2)	C8—N7—C6—O18	−177.9 (2)
C16—N1—C9—C4	179.9 (2)	C10—N7—C6—O18	2.0 (3)
C2—N1—C9—C8	177.9 (2)	C8—N7—C6—N5	1.3 (3)
C16—N1—C9—C8	−2.0 (4)	C10—N7—C6—N5	−178.80 (19)
C2—N3—C4—C9	0.1 (3)	C12—C13—C14—O20	8.2 (3)
C2—N3—C4—N5	−179.1 (2)	C12—C13—C14—C15	−171.5 (2)
N1—C9—C4—N3	0.1 (3)	C14—C13—C12—C11	178.31 (19)
C8—C9—C4—N3	−178.2 (2)	C6—N7—C8—O19	179.1 (2)
N1—C9—C4—N5	179.3 (2)	C10—N7—C8—O19	−0.8 (3)
C8—C9—C4—N5	1.0 (4)	C6—N7—C8—C9	−1.5 (3)
C6—N5—C4—N3	177.8 (2)	C10—N7—C8—C9	178.54 (19)
C17—N5—C4—N3	−3.8 (4)	C4—C9—C8—O19	179.7 (2)
C6—N5—C4—C9	−1.3 (3)	N1—C9—C8—O19	1.8 (4)
C17—N5—C4—C9	177.1 (2)	C4—C9—C8—N7	0.4 (3)
C6—N7—C10—C11	87.7 (2)	N1—C9—C8—N7	−177.5 (2)
C8—N7—C10—C11	−92.3 (2)	N7—C10—C11—C12	−175.8 (2)
C4—N5—C6—O18	179.4 (2)	C13—C12—C11—C10	71.4 (3)
C17—N5—C6—O18	1.0 (3)	C4—N3—C2—N1	−0.3 (3)
C4—N5—C6—N7	0.2 (3)	C9—N1—C2—N3	0.3 (3)
C17—N5—C6—N7	−178.2 (2)	C16—N1—C2—N3	−179.8 (2)

### Hydrogen-bond geometry (Å, °)

**Table:**

*D*—H···*A*	*D*—H	H···*A*	*D*···*A*	*D*—H···*A*
C2—H2···O18^i^	0.93	2.39	3.206 (4)	147
C15—H15B···O19^ii^	0.96	2.60	3.439 (4)	147
C16—H16A···O18^i^	0.96	2.55	3.395 (4)	148

Symmetry codes: (i) −*x*−1, *y*+1/2, −*z*−1/2; (ii) −*x*−2, *y*−1/2, −*z*−1/2.
